# Draft Genome Sequence of *Lactobacillus plantarum* 2165

**DOI:** 10.1128/genomeA.01179-13

**Published:** 2014-01-09

**Authors:** Andrey V. Karlyshev, Vyacheslav M. Abramov

**Affiliations:** School of Life Sciences, Faculty of Science, Engineering and Computing, Kingston University, Penrhyn Road, Kingston upon Thames, United Kingdoma; Institute of Immunological Engineering, Lyubuchany, Moscow Region, Russiab

## Abstract

This report describes a draft genome sequence of *Lactobacillus plantarum* 2165. The data demonstrate the presence of a large number of genes responsible for sugar metabolism and the fermentation activity of this bacterium. Different cell surface proteins, including fibronectin and mucus-binding adhesins, may contribute to the beneficial probiotic properties of this strain.

## GENOME ANNOUNCEMENT

Various strains of *Lactobacillus plantarum* have been found to possess a wide range of beneficial properties useful both in the food industry and in medicine. One example is the antifungal activities of some strains of this species (1).

*L. plantarum* strain 2165 was isolated from a soured milk product in 2011 at the Institute of Engineering Immunology, Russia. A remarkable feature of this strain is its ability to ferment various vegetable juices, including beetroot juice. The strain is deposited at the All-Russian Collection of Microorganisms at the GK Skryabin Institute of Biochemistry and Physiology of Microorganisms (Russian Academy of Sciences, Pushchino, Russia) under the registration number VKM B-2725D.

Here, we present a draft genome sequence of *L. plantarum* strain 2165 generated by using the Ion Torrent PGM. At the time of preparation of materials for this publication, six complete *L. plantarum* genome sequences were available (strains 16, WCFS1, JDM1, ZJ316, P-8, and ST-III). All but one of these strains contain plasmids (between 1 and 10). Genome sequencing of *L. plantarum* 2165 resulted in 448,592 reads, with a total size of 59.8 million bases. Assembly of the reads using the Ion Torrent assembler plugin produced 192 contigs (0.5 to 120 kb; mean size, 16.5 kb). The total size of the assembly (3,179,972 bases, 18.8× genome coverage) and G+C content (44.5%) match the figures for the published genome sequences of other strains of this species (3.2 to 3.36 Mb and 44.4 to 44.7%, respectively).

The mapping of reads onto the sequences of the reference strains using the CLC Genomics Workbench program revealed low similarity of the plasmids. A total of 88.5% of the reads were mapped onto the genome of strain *L. plantarum* WCFS1 (2). Among the features present in the latter but missing in the test strain were the genes encoding a mannose-specific adhesin, various phosphotransferase systems (PTS), CDP glycerol phosphotransferase, and a type I restriction modification system. A number of genes in the capsular polysaccharide cluster were also missing, suggesting the presence of a capsule with a structure different from that in strain WCFS1. A BLASTx search using the assembled contigs identified a number of cell surface protein precursors, in addition to six mucus and two collagen-binding adhesins. In addition, a plantaricin biosynthesis gene cluster was detected.

NCBI GenBank annotation of the *L. plantarum* 2165 genome resulted in the identification of 3,141 genes encoding 2,702 proteins, while the RAST server (3) detected 3,100 protein-coding sequences. Among the remarkable features detected is a potential to produce teichoic acid, shown to have an immunostimulatory activity (4), and the presence of genes required for resistance to bile salts, tetracycline, fluoroquinolones, and other toxic compounds, as well as those involved in the biosynthesis of multidrug resistance efflux pumps. A large number of genes (412) are associated with sugar metabolism.

The availability of the genome sequence of *L. plantarum* 2165 will assist in better understanding of the factors responsible for the beneficial features of this microorganism and may assist in the development of novel probiotics.

### Nucleotide sequence accession numbers.

This whole-genome shotgun project has been deposited at DDBJ/EMBL/GenBank under the accession no. AVFI00000000. The version described in this paper is version AVFI01000000.
